# Sero-epidemiological survey of human cytomegalovirus-infected children in Weifang (Eastern China) between 2009 and 2012

**DOI:** 10.1186/1743-422X-10-42

**Published:** 2013-02-01

**Authors:** Xiuning Sun, Zhijun Liu, Bin Wang, Lihong Shi, Ruiwen Liang, Ling Li

**Affiliations:** 1 Department of Microbiology, Key Laboratory of Medicine and Biotechnology of Qingdao, Qingdao University Medical College, 266071, Qingdao, Shandong, China; 2 Department of Parasitology, Weifang Medical University, 7166 Baotong West Street, 261053, Weifang, Shandong, China; 3 Department of Medical Microbiology, Weifang Medical University, 7166 Baotong West Street, 261053, Weifang, Shandong, China; 4 Department of Pharmacology, Weifang Medical University, 7166 Baotong West Street, 261053, Weifang, Shandong, China

**Keywords:** HCMV, Serology, Epidemiology

## Abstract

**Background:**

To understand the prevalence and characteristics of human cytomegalovirus (HCMV) infection in children in the Weifang area, and to provide information for its prevention and treatment.

**Methods:**

A comprehensive survey was performed from 2009 to 2012 in 7582 children from birth to 6 years of age hospitalized in the Maternity and Child Health Hospital of Weifang. ELISA HCMV serology results and survey data were analyzed by age group and socio-economic level. The infection rates were based on IgG and IgM serology.

**Results and conclusions:**

The overall infection rate from IgG and IgM in the Weifang area from 2009 to 2012 was 42.5% (3496/7582), among which 34.2% were HCMV-IgG positive, suggesting past infection. Overall, the probability of active HCMV infection showed no gender difference in any age group (P >0.05). Recent infections centered on the first 6 months of life, presumably due to breastfeeding. Among the 654 children hospitalized for active HCMV infection, 379 (57.9%) were from rural areas and 275 (42.1%) from urban areas, showing that active infection in the countryside was higher than that in the city (χ^2^ = 32.65, p <0.01).

## Introduction

Human cytomegalovirus (HCMV) belongs to the beta herpes family and it is one of the most common causes of human infectious diseases. Most people acquire the infection in childhood or youth. HCMV infection often affects multiple systems; while liver involvement is more common [1], it can also cause problems in the respiratory, nervous, and vascular system, and lead to fetal malformations [2-5].

Sero-epidemiological studies show that in developed countries such as the UK and the USA, ~50% of adults show evidence of past HCMV infection [6]. In developing countries, HCMV infection in older children and adults can be as high as 90-100%. Epidemiological studies play an increasingly prominent role in its prevention and prognostic evaluation. Therapy can only effectively suppress viral replication, but cannot completely eliminate the virus. So far, no clinically safe and effective HCMV vaccine is available. Here, we carried out a survey over the past three years in Weifang, a city in eastern China, to provide a database for HCMV infection in children so as to better understand the epidemiological characteristics and clinical features.

## Results and discussion

### Total serology results

Among the 7852 children, the overall rate of infection was 42.5% (overall % = IgM^+^ % + IgG^+^ IgM^+^ % + IgG^+^ %). The results are detailed in Table 1.

**Table 1:** Serological results from 7852 children with HCMV in Weifang from 2009 to 2012

**HCMV-IgG**	**HCMV-IgM**	**Number of cases (%)**
-	-	4516 (57.5)
-	+	311 (3.9)
+	+	343 (4.4)
+	-	2842 (34.2)

### Age distribution

**Figure 1:**
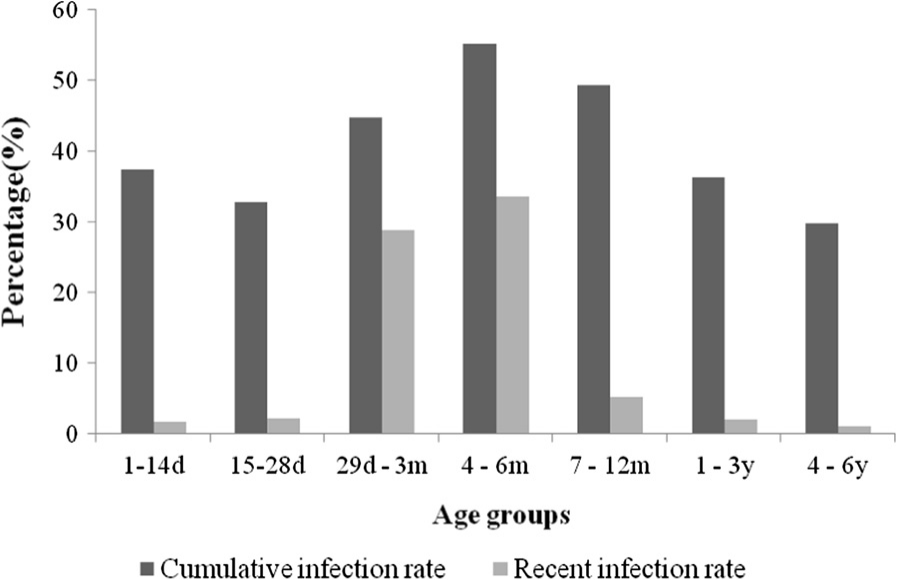
Total and recent infection rates in different age groups.

The total infection rate peaked in the 4–6 month age group. The data analysis showed that recent infections centered on babies aged 29 days to 6 months (Table 2, Figure 1).

**Table 2:** Distribution of serological results in different age groups

**Age group**	**IgG**^**- **^**IgM**^**- **^**(%)**	**IgG**^**+ **^**alone (%)**	**IgM**^**+ **^**alone (%)**	**IgG**^**+ **^**IgM**^**+ **^**(%)**	**Overall IgM**^**+**^	**Total**	**Cumulative infection rate**	**Recent infection rate**
**1**-**14 d**	938	548	8	8	16	1502	37.5	1.7
	(62.5)	(36.5)	(0.5)	(0.5)	(1)	(100)		
**15**-**28 d**	384	179	8	1	9	572	32.9	2.3
	(67.1)	(31.3)	(1.4)	(1.7)	(1.5)	(100)		
**29 d**-**3 m**	982	392	221	180	401	1775	44.7	28.9
	(55.3)	(22.1)	(12.5)	(10.1)	(22.6)	(100)		
**4**-**6 m**	315	230	50	110	160	705	55.3	33.7
	(44.7)	(32.6)	(7.1)	(15.6)	(22.7)	(100)		
**7**-**12 m**	944	868	17	35	52	1864	49.3	5.2
	(50.6)	(46.6)	(0.9)	(1.9)	(2.8)	(100)		
**1**-**3 y**	521	288	6	5	11	819	36.4	2.1
	(63.6)	(35.1)	(0.7)	(0.6)	(1.3)	(100)		
**4**-**6 y**	432	178	1	4	5	615	29.8	1.1
	(70.2)	(29.0)	(0.2)	(0.6)	(0.8)	(100)		
**Total**	5048	2836	315	346	661	7852		

Note: Cumulative infection rate % = (total number of age group - both neigative number)/total number × 100.

Recent infection rate % = overall IgM^+^ number of the age/(total number of the age - IgG^+^ number) × 100.

### Regional distribution of active HCMV infection

In 7852 children, 654 (8.3%) were serum IgM-positive, indicating active HCMV infection. Among the 654 children hospitalized with active infection, 379 (57.9%) were from rural areas while 275 (42.1%) were from urban areas. The difference was significant (χ^2^ = 32.65, p <0.01).

## Discussion

HCMV seroepidemiology is determined by factors such as age and socio-economic/public health conditions, and varies from 40% to 100% seropositive adults in different geographic and socio-economic populations. According to the epidemiological data obtained in 2009, the HCMV seroprevalence in Ji'nan, China, is 33.49% in people <20 years old while it is 48.3% in those >20 years old [7]. Our seroepidemiological survey showed that the overall infection rate of HCMV in children from 2009 to 2012 was 42.5%, higher than that in Ji'nan. Compared to other countries, the rate was lower than in African populations <10 years old, but higher than populations in developed countries <15 years old. In our data, the HCMV-IgG-positive rate was 34.2%, suggesting serological evidence of past infection; this is a lower positivity-rate than in developed countries. However, in infants <6 months old, it is unclear whether this was due to vertical transmission or fetal transmission from the mother.

A clinical study of HCMV reporting 186 hospital cases from 2003 to 2007 showed that the onset age of HCMV infection is usually within the first year [7], more in males than in females (1.9:1). From our survey, new HCMV infections were concentrated in infants <6 months old, which is consistent with previous reports.

Our data showed that the total infection rate was significantly different between age groups (except for 15–28 days) and 6 months seemed to be the threshold. Ages under 6 months patients due to the impact of mother transmitted IgG antibody and gradually increased recent infection rate, the cumulative infection rate was the highest until 6 months. Nevertheless, after 6 months a decreased trend was seen. The reason might be that the IgG titers decrease gradually with increasing age.

In addition, the statistical analysis showed that both the overall distribution and active HCMV infection did not different by gender in children in Weifang (p >0.05). It is known that HCMV can be transmitted to the newborn *via* the birth canal, breast milk or other sources. In preterm infants, who are easily infected by HCMV, the acquired infection ranges from 12–22%, depending on the gestational age and the breastfeeding duration and frequency as well as other factors [8]. Thus, although breast milk is recognized to be the ideal feeding method for newborns, it can transmit HCMV to the offspring of HCMV-seropositive mothers. A seroepidemiologic study in Taiwan indicated that the duration of breastfeeding is a significant risk factor for HCMV seropositivity. Children given HCMV-seropositive breast milk for >24 months are 2.43 times more likely to be infected than those who are breast fed for ≤24 months (p = 0.0001) [9]. Heating and freezing procedures can prevent infection *via* breast milk in preterm infants [10,11]. While these procedures might not completely eliminate HCMV, they are less harmful to the immunological factors contained in breast milk [12,13].

Seroepidemiological studies also showed that in developed countries, the HCMV antibody-positive rates in the high-level socio-economic population is 40-60% while in the low-level population the rate is >80% [14]. According to one analysis, the HCMV infection ratios in low-, medium- and high-income families are 3.5:2.1:1.5 [15]. In our survey, 57.9% of the HCMV-IgM-positive hospitalized children were from rural areas and 42.1% from urban areas, suggesting economic conditions can greatly affect HCMV infection.

It should be noted that the screening of patients for HCMV infection is generally performed using either competitive or indirect ELISAs which detect both IgG and IgM antibodies. A major disadvantage of HCMV IgG/IgM antibody detection is the relatively high frequency of false-positives which may be observed in the absence of specific IgG antibody [16]. False-positive reactions occur in various conditions unrelated to active HCMV infection, including polyclonal antibody production during infections with other herpes viruses [17], Epstein-Barr virus, and autoimmune disease [18-20]. In this study, we excluded other possible infections. Limitations of this survey are that all patients needed to be screened for the presence of CMV-DNA, especially late gene transcripts in their blood or urine samples by using nested-PCR [21]. Despite its preliminary character, this study clearly indicates that recent HCMV infection is centered on the first 6 months of life with the evidence from a large population sample; and the details of breast milk from HCMV IgM-positive mothers as a source of infection in children <6 years old remain to be clarified.

## Conclusions

In summary, a sero-epidemiology survey was conducted in Weifang, China. The overall infection rate is 42.5%. Understanding HCMV prevalence in this eastern city provides valuable information regarding the epidemiology of HCMV. Protection against this infection needs greater emphasis.

## Methods

### Patients and grouping

Between January 15, 2009 and January 15, 2012, 7852 serum samples were obtained from patients in the Maternity and Child Health Hospital of Weifang. The parents of all subjects gave written informed consent to the study protocol, which was approved by the Ethics Committee of Weifang Medical University (reference number, wfmu2009005). After excluding those infected with *Toxoplasma gondii*, rubella virus, *Herpes simplex* virus-1, hepatitis virus or syphilis, the participants were divided into 7 sub-groups based on age: 1–14 days, 15–28 days, 29 days–3 months, 4–6 months, 7–12 months, 1–3 years and 4–6 years. Data were recorded as follows: negative for both CMV-IgG and IgM, positive for HCMV-IgG alone, positive for IgM alone, positive for both IgG and IgM, and overall positive for IgM.

### Serology and statistics

HCMV serology was performed in the Central Clinical Laboratory, the Maternity and Child Health Hospital of Weifang, from January 2009 to January 2012. The presence of HCMV-specific IgM and IgG antibodies was assayed using ELISA (Quida, USA), according to the manufacturer’s instructions. The plate washer (Stat Fax 2600) and microplate reader (Stat Fax 2100) were from Awareness Technology, Inc., (USA). The sample-to-cutoff rate (S/CO) was calculated to provide the numeric value of the test. An S/CO value >1 is considered as positive, according to the manufacturer. All information was entered into Excel to create a database and SPSS 13.0 was used for the χ^2^ test; p <0.05 was considered statistically significant.

## Abbreviations

HCMV: Human cytomegalovirus; ELISA: Enzyme-linked immunosorbent assay.

## Competing interests

The authors declare they have no competing interests.

## Authors’ contributions

XS, ZL and BW contributed to the design of this study; XS, LS and BW contributed to the data collection and analysis; RL and LL contributed to the data analysis and statistics; XS, ZL and BW contributed to the manuscript writing and its final approval. All authors read and approved the final manuscript.
